# Beyond Gisin’s Theorem and its Applications: Violation of Local Realism by Two-Party Einstein-Podolsky-Rosen Steering

**DOI:** 10.1038/srep11624

**Published:** 2015-06-25

**Authors:** Jing-Ling Chen, Hong-Yi Su, Zhen-Peng Xu, Yu-Chun Wu, Chunfeng Wu, Xiang-Jun Ye, Marek Żukowski, L. C. Kwek

**Affiliations:** 1 Theoretical Physics Division, Chern Institute of Mathematics, Nankai University, Tianjin 300071, People’s Republic of China; 2 Centre for Quantum Technologies, National University of Singapore, 3 Science Drive 2, Singapore 117543; 3 Key Laboratory of Quantum Information, University of Science and Technology of China, 230026 Hefei, People’s Republic of China; 4 Synergetic Innovation Center of Quantum Information and Quantum Physics, University of Science and Technology of China, 230026 Hefei, Anhui, China; 5 Pillar of Engineering Product Development, Singapore University of Technology and Design, 8 Somapah Road, Singapore 487372; 6 Institute of Theoretical Physics and Astrophysics, University of Gdańsk, PL-80-952 Gdańsk, Poland; 7 Hefei National Laboratory for Physical Sciences at Microscale and Department of Modern Physics, University of Science and Technology of China, 230026 Hefei, China; 8 National Institute of Education,1 Nanyang Walk, Singapore 637616; 9 Institute of Advanced Studies, Nanyang Technological University, 60 Nanyang View, Singapore 639673

## Abstract

We demonstrate here that for a given mixed multi-qubit state if there are at least two observers for whom mutual Einstein-Podolsky-Rosen steering is possible, i.e. each observer is able to steer the other qubits into two different pure states by spontaneous collapses due to von Neumann type measurements on his/her qubit, then nonexistence of local realistic models is fully equivalent to quantum entanglement (this is not so without this condition). This result leads to an enhanced version of Gisin’s theorem (originally: all pure entangled states violate local realism). Local realism is violated by all mixed states with the above steering property. The new class of states allows one e.g. to perform three party secret sharing with just pairs of entangled qubits, instead of three qubit entanglements (which are currently available with low fidelity). This significantly increases the feasibility of having high performance versions of such protocols. Finally, we discuss some possible applications.

Quantum mechanical correlations do not admit local realistic models. This pivotal result concerns the foundations of quantum mechanics (QM) and has many applications in quantum information theory. The states exhibiting quantum correlations can be grouped using the following hierarchy^1^: those that are entangled, those that allow for Einstein-Podolsky-Rosen (EPR) “steering”, and those that violate local realism (LR)^2^.

According to Erwin Schrödinger^3^, quantum entanglement is “the characteristic trait of quantum mechanics” distinguishing it from any classical theory. This feature of quantum states is a highly useful resource in many fascinating applications of quantum information, such as teleportation^4^,^5^, dense coding, communication protocols, and computation^6^,^7^. Moreover, states that violate local realism have gained ubiquitous applications in different quantum information tasks, such as quantum key distribution^8^, communication complexity^9^, quantum information processing and random number generation^10^.

In Schrödinger’s reply to the EPR paper^11^, he made a fine distinction between entangled states and states shared between two parties that are amenable for steering, i.e. in which the action of one party can affect the reduced state of the other party through the an appropriate choice of measurements. Also, in contrast to quantum entanglement which has received widespread interest due to its usefulness as a resource for tasks in quantum information processing, there have been relatively fewer developments in the notion of “steerable” states. In 2007, Wiseman *et al.*^1^ revisited the issue and reformulate the idea of “steerable” state from a quantum information perspective. Since then, several interesting studies on EPR steering have appeared both in theory^12^,^13^ and in experiments^14^,^15^,^16^,^17^,^18^. Recently, some of us have demonstrated an all-versus-nothing proof of EPR steering^19^.

Quantum steering in a bipartite scenario essentially describes the ability of one party, say Alice, to prepare the other party’s (Bob’s) systems in different ensembles of quantum states by measuring her own particle (under more than two different settings). Naturally, Alice has no control over the actual result of her actions, but she is able to control over the set of projected states on Bob’s side with her measurement for a given setting. If Bob does not trust Alice, Alice’s manipulation of her system appears as a black box described by some local hidden variable (LHV) theory. This could imply a local hidden state (LHS) description of her actions: that is that she just simply sends some states to Bob according to some probability distribution. Quantum steering means that such a description is impossible and that Alice must use quantum measurements on her system to prepare states on Bob’s side.

As pointed by Wiseman *et al.*^1^, there is a hierarchical structure. For a given state, quantum steering is strictly implied by the violation of local realism. Simply put, steering excludes the possibility of local “hidden” state models of correlations, in which a quantum mechanical model is applied to only one of the systems, while the other one is described using a local hidden variable model. LHS model allows for a full LHV model for both (all) systems. Of course separable (mixed) states can be thought of as probabilistic distributions of hidden states for each party, and thus states with steering are a proper subset of entangled states, endowed with an additional potentially useful property.

The close connection between quantum entanglement and violation of local realism^20^ can be traced back to Gisin’s work, Ref. 21, in which he presented a theorem stating that any pure entangled state of two qubits violates a Bell-like inequality. The result was generalized in Refs. 22,23. Gisin’s theorem for three qubits was shown numerically by Chen *et al.*^24^ and analytically by Choudhary *et al.*^25^. In 2012, Yu *et al.*^26^ provided a complete proof of Gisin’s theorem for all entangled pure states. Within the hierarchical picture of the states exhibiting quantum correlations, Gisin’s theorem says that violation of local realism and quantum entanglement are equivalent for all *pure* states.

Gisin’s theorem applies only to pure states. The aim of this paper is to develop an “enhanced” version of Gisin’s theorem that can apply also to some class of mixed states. We shall show that some form of EPR steering allows the Gisin’s theorem to be applicable to a wider range of entangled states than just pure ones. As pure entangled states always allow steering, we have a direct broadening of the realm of validity of Gisin’s theorem. Our result also provides a rigorous criterion for marking the borders between quantum entanglement, EPR steering and violation of local realism. This is a nontrivial problem since it is not easy to reduce a superset to a subset by imposing extra constraints.

Our result serves as a contribution to recent studies of general nonlocal theories which satisfy the non-signaling principle^27^,^28^ (or its extensions^29^), asking the question under what conditions an entangled state does not violate local realism^30^. Other links are with studies of the role of quantum contextuality in violation of local realism like e.g. Ref. 31. In Methods, we prove two theorems regarding the EPR steerability. We also note that the original Gisin’s theorem is a special case of Theorem 1. We note that our criterion for steerability of quantum states is useful in some applications. We then describe how our criterion for steerability of quantum states could be applied to the Third Man cryptographic protocol and we show how Theorem 2 can serve as a valuable resource in a quantum certificate authorization protocol.

## Results

### Enhanced Gisin’s theorem

Gisin’s original work starts with two qubits in a pure state. In the spirit of this, let us also start with the two-qubit case, for which we have the following theorem:

**Theorem 1** *For a two-qubit entangled state,* 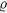
*, shared between Alice and Bob, the state violates local realism if any party can steer the other party into two different pure states by performing on her/his qubit some orthogonal projective measurements* 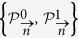
*, where* 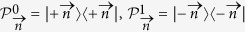 *are projectors along the* 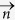
*-direction.*

Note that in the theorem, the word “different” refers to the fact that the reduced states of one party (after the other performs the measurements) are different.

*Proof*. Take the spectral decomposition of two-qubit density matrix: 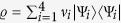, the positive *ν*_*i*_ add up to one. For 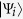, if Alice performs the orthogonal projective measurements on on her qubit given by 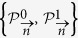 along the 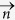-direction and is able in this way to steer Bob’s qubit into two different states 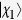 and 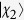, then the state 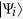 must be in the form


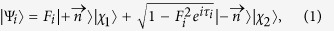


with real *F*_*i*_ satisfying 0 < *F*_*i*_ < 1. However, in the formula we have only two mutually orthogonal vectors 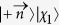, 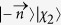. This means that we can have only the two independent 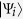 with the same steer_*i*_ng property. Hence, the rank of 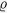 is at most 2:


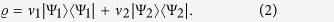


If also Bob with measurements on his qubit can steer Alice’s qubit into two different pure states, this additionally constrains the form of the two pure states to


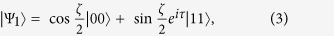



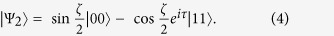


For a derivation see Methods

A mixture of the such states, with *ν*_1_ ≠ *ν*_2_, always violates the Clauser-Horne-Shimony-Holt (CHSH) inequality. In quantum mechanics the correlation function is computed using 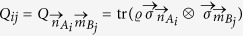, where 
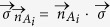
, and 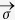; is the Pauli matrix vector, whereas 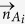 is the *i*-th measuring direction of Alice. We shall use the spherical coordinates, so that 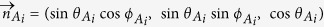; similarly for Bob. The local realistic CHSH constraint is that 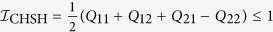. By putting 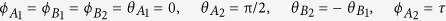, we get 
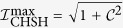
, where 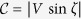 is the degree of entanglement, and *V* = *ν*_1_ − *ν*_2_. Except at *ζ* = 0, π or *V* = 0, 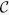 is always nonvanishing, and the CHSH inequality is violated.

Note that other criterions for steerability, e.g., the steerable weight proposed in Ref. 32 as a measure to effectively quantify the EPR steering, could also be used to prove the theorem; however, developing a stricter steering criterion is beyond our aim of the present paper. Note also that, the original Gisin’s theorem is a special case of Theorem 1. For example, take *ν*_1_ = 1. The state 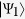, for *ζ* ≠ 0 or π, is a two-qubit pure state in its Schmidt decomposition, and its steerable weight equals 1. Moreover, when *ν*_1_ = *ν*_2_ = 1/2 (i.e., *V* = 0), the state becomes a separable state, that has a vanishing degree of entanglement and does not violate any Bell inequality.

For the general case of *N* qubits, we have the following theorem:

**Theorem 2** *For an N-qubit entangled state shared by N observers* 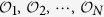
*, the state violates local realism if there exists at least two observers, each with the ability to steer the remaining N* *−* *1 qubits into two different pure states by performing on her/his qubit some orthogonal projective measurements.*

Note that as in **Theorem 1**, “different” refers to the fact that the reduced states of the remaining *N* − 1 parties (after one observer, say 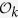, performs his measurements) are different.

Without any loss of generality, we assume that the first two observers have the ability to steer the remaining *N* − 1 qubits. As such, the state 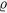 is either (a) an arbitrary *N*-qubit entangled pure state or (b) a rank-2 density matrix as in (2) with


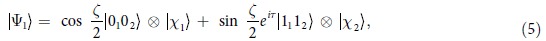



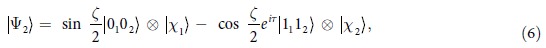


states for 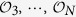, and the relative phase *τ* can always be taken as zero.

The violation of local realism for Case (a) has been shown analytically in^25^,^26^ with the use a generalized Hardy (see e.g. inequality^33^,^34^,^35^). To prove Case (b), it is convenient to consider first *N* = 3, before moving to *N* ≥ 4. (See Methods for the rigorous proof).

### Application 1: The Third Man cryptography

We have extended the class of states for which Gisin’s Theorem holds. But are these new states endowed with properties that can be put to use in quantum information tasks, do they form a kind of new resource? Below we shall show that in specific cases they can reduce the number of entangled particles needed to perform a task. In our example for three partners this is The Third Man cryptography^36^,^37^ or equivalently secret sharing^38^. The example is extendible to more partners.

Imagine that Charlie is sending the states of Theorem 1 to Alice and Bob. He randomly chooses whether to send 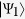 or 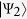, with probabilities *ν*_1_ and *ν*_2_, which are different but quite similar. To simplify the example, assume that both pure states are maximally entangled, that is *ζ* = π/2. Alice and Bob are asked to perform randomly chosen measurements using local bases 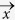 and 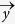, and also in some auxiliary bases in the *xy* planes, usually needed in the Ekert91 protocol^8^. But the trouble is that while one of the pure states gives perfectly correlated results when both measured in 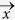 direction, and anti-correlated results for measurements in directions 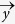, the other one gives perfect anti-correlations for 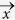 measurements and perfect correlations for 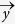
’s (it is irrelevant for the argument for which of the states this is so). Thus only if Charlie sends them information about which state he sent in the given run, they can unscramble the key, out of their measurements in the identical local bases. Thus Charlie holds a key to their key. Without additional information provided by him Alice and Bob cannot form a usable key. This is the Third Man cryptography.

Of course, if Charlie sends information on the states to just Alice, she can transform her string of data into one which is a copy of the string of Bob, and she can also build a working key (this is a secret sharing version of the same protocol). Of course, all this is done under the standard Ekert91 protocol, Alice and Bob exchange information on measurements bases without revealing the results, etc. Note, that for high, say *ν*_1_, with classical error correction methods Alice and Bob could be able to extract a key, but it will be short, with respect to the numbers of runs (copies of the state sent by Charlie). Only with the help of Charlie it can be of maximal possible length (half of the numbers of runs, if the protocol runs perfectly).

Previous versions of such protocol, see^36^,^37^ or^38^, required three qubit entangled GHZ states. The reader may quickly judge how difficult the step is from two-particle entanglement distribution to three-particle entanglement distribution by consulting the review^7^. Also the fidelity of photonic three-particle entangled states is currently still typically below 90%, while in the two-particle case it can be now well over 99%. Low fidelity leads to errors in the key distribution. Thus the scheme with steerable states has a clear advantage over the the original one with GHZ states.

There is one more advantage. By making measurements in the 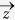 directions, which are always perfectly correlated, Alice and Bob can check whether the mixed state received by them from Charlie (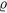, that is the state before he reveals in which runs were 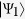 or 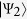) is indeed entangled (as it violates the CHSH inequality). Thus they can have an independent quality check of Charlie’s distribution methods. This is impossible under GHZ stated based protocols.

Note that the protocol settings for Alice and Bob are 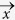 and 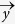. With settings in 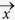 and 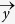 Alice and Bob cannot violate the CHSH inequality with 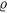 for which *ν*_1_ and *ν*_2_ are close. Using the Horodecki criterion for violations of the CHSH inequality^39^, 
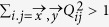
, given here in the form derived in^40^, one can easily establish that the threshold difference is 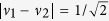. The correlations in these directions are very weak, thus they are indeed unable to extract a key with such measurements, and the Ekert91 protocol. Without Charlie’s help they are helpless. Of course, the presented scheme can be modified in many ways.

### Application 2: The quantum certificate authorization protocol

The state 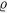 we discussed in **Theorem 2** can serve as a valuable resource in quantum cryptography^41^. A good example is an application to quantum certificate authorization involving three parties, say, Alice, Bob and Charlie, against a lurking eavesdropper, Eve. Suppose Alice needs to send some private information to Bob through internet, and yet she is not sure if the receiving party is Bob. So Alice goes to the certificate authority, Charlie, who is trusted by Alice and capable of certifying Bob’s identity. With Charlie’s help, Alice is then able to share secret keys with Bob at a distance.

**Figure 1:**
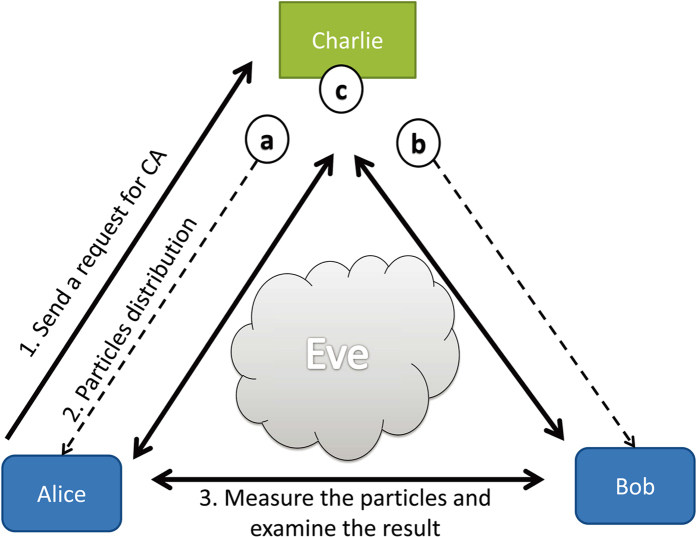
(Color online) The illustration of quantum certificate authorization protocol. Upon Alice’s request for identification of Bob, Charlie produces a three-qubit state 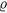 and then distributes the first qubit (represented as a ball labeled by “a”, similarly for the others) to Alice , the second qubit “b” to Bob, and keeps the third qubit “c”. To ensure the security, Charlie randomly measures his qubit along 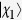 or 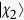, Alice and Bob randomly measure their qubits along 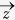 or 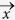. Such a measurement can be repeated for large enough times. Finally Charlie performs a random inspection to see whether Alice and Bob are able to share secret keys at the quantum level that defy Eve’s eavesdrop.

There are two goals to be achieved here. Specifically, Alice’s private information (i) should be received by the true Bob (and not someone else who claims to be Bob) and (ii) should not be intercepted by Eve. This task can be classically realized through digital signatures and public-private keys. Given 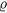, we see that we are able to present a quantum analogue of this protocol (see also^42^,^43^). As shown in Fig. 1, upon Alice’s request for identification of Bob, Charlie produces an ensemble of three-qubit states 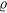
’s and distributes the first qubit to Alice, the second qubit to Bob, and keeps the third qubit. Alice and Bob measure their qubits randomly along one of two directions: 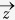 or 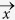 (i.e., projections to 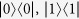 or 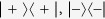); similarly Charlie also measures his qubit randomly by a projection to 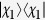 or 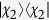. Such a joint measurement can be repeated for, say, *N* times, where *N* is large enough. If the state shared by Alice and Bob is reliable, they should get the same results (cf. Eqs. (4) in the main text) for any measurement along 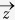.

In order to detect a possible Eve, Charlie randomly picks *m* runs from *N* as a subset and requests Alice and Bob to broadcast through a public channel their directions and results for these *m* runs. With their data at hand Charlie starts to do analysis in three steps: (i) keep joint measuring results for which Alice and Bob measured along the same direction, i.e., 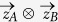 and 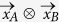, and discard those for 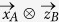 and 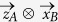; (ii) keep joint measuring results for which Charlie measured along 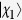 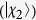 when Alice and Bob obtained both “0” (“1”) along 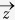; (iii) verify (a) whether Alice (Bob) can steer Bob (Alice) and Charlie into two pure states when measuring along 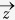, and (b) whether *V* sin *ζ* ≠ 0.

Here (a) can be verified by examining whether Charlie’s probability of obtaining 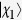 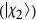 is always unity when Alice and Bob obtained both “0” (“1”) along 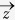. For (b), the three-qubit state 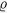 is entangled iff *V* sin *ζ* ≠ 0. To realize the quantum certificate authorization protocol, Charlie can produce 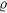 with nonorthogonal 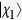 and 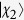. The coincidence probability that Alice and Bob get the same result can be obtained as 
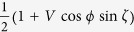
 when Alice and Bob measure along 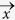. To check whether 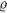 is entangled is equivalent to check whether the coincidence probability is not equal to 1/2. In other words, result (a) ensures that both Alice and Bob can steer the remaining parties into two pure states; and if (a) is true, result (b) certifies that the state 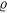 is entangled. Hence, if both (a) and (b) are fulfilled, then according to **Theorem 2** the state 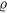 violates local realism, and the protocol is secure. Note that the secret keys are obtained from the unbroadcast part of results when Alice and Bob measure along 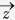.

## Discussion

Our result sheds new light on the relation between entanglement of mixed states, and LHV models for correlations. It pinpoints a precisely defined class of states, which is strictly larger than all pure entangled states, and which has the property that just by the fact that a state belongs to the class, one knows that it violates local realism. The property is as follows: for an *N*-qubit state, there exist a pair of observers such that each of them singlehandedly can steer the remaining *N* − 1 qubits of the other observers into two different pure states. Our results are for qubit systems. A generalization to more complicated ones is still an open question, and under investigation. Mixed states covered by the enhanced Gisin theorem, due to their specific properties, may allow new quantum protocols which allow to reduce the complication of entangled states involved in them (as in our example, we need, e.g., two-particle entangled states to have secret sharing between three parties). Other protocols, such as quantum certificate authorization, are also possible.

## Methods

### Proof of Eqs. (3) and (4)

Without loss of generality, if Alice measures her qubit in the *z*-direction and if she steers Bob’s qubit into two different pure states, then for the states (2) we have


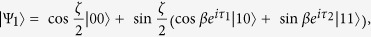



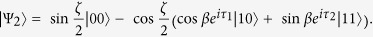


However, if Bob measures his qubit along some specific direction, also Alice should be getting one of two steered states. We can denote Bob’s projectors by 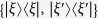. The respective qubit basis states can be put as


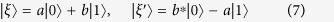


with *a* real-valued, 
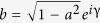
. If the state of a qubit is pure, then the determinant of its density matrix is zero. If Bob measures his qubit of 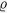, given by (2), with the above projectors, the determinants of the steered states of Alice’s qubit are


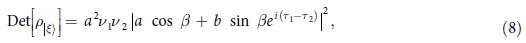



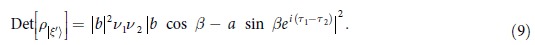


We must find conditions for both of them to vanish. For the only interesting case of both *ν*_*i*_ greater than zero, we have to consider two situations. (i) Suppose *a* = 0, then it is enough to have 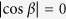. (ii) Suppose *a* ≠ 0, then one has the conditions 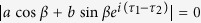 and 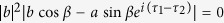. The only solution is *a* = 1 (i.e., 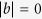) and 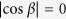. This directly leads to (3) and (4). For the general *N* qubits, one has a straight ahead extension of this reasoning leading to Eqs. (5) and (6).

### Proof of Theorem 2 for *N* = 3

Take three observers Alice, Bob and Charlie. Assume that Alice and Bob have the ability to steer the remaining two qubits. Due to local unitary, one can always work with 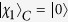, 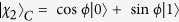, and 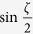, 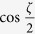, sin*ϕ*, cos*ϕ* ≥ 0.

(i) Let us first consider the case where cos*ϕ* ≠ 0. The three-qubit Hardy inequality is given by


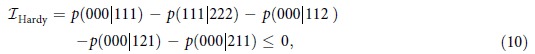


where 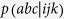 denotes the probability *p*(*A*_*i*_ = *a*, *B*_*j*_ = *b*, *C*_*k*_ = *c*). Let the settings be 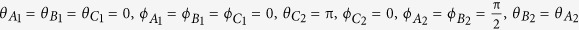, the quantum prediction then becomes


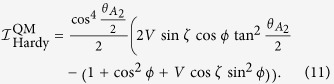


By taking


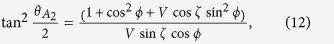


we finally have


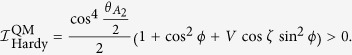


(ii) If cos*ϕ* = 0, we employ the following Bell inequality:


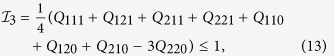


where *Q*_*ijk*_ are the qubit correlation functions defined in analogy to the two qubit ones (see above). The index 0 indicates that measurement performed on the corresponding qubit do not enter the function, and thus we have a two-qubit correlation function. By taking settings as 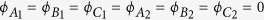, 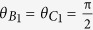, 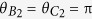, and 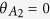, all correlation functions vanish except the following ones: 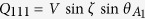, 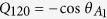, and *Q*_220_ = −1. The quantum prediction becomes


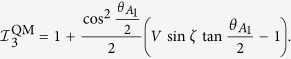


Clearly, we have 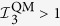 when 
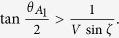


### Proof of Theorem 2 for Cases *N* ≥ 4

For *N* ≥ 4, the states 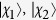 can be entangled. The proof can be split into two cases: (i) separable 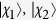, and (ii) at least one of them being entangled. To demonstrate violations of local realism, we employ an *N*-qubit Hardy inequality, together with some Bell inequalities devised by us particularly for the present paper.

The *N*-qubit Hardy inequality reads


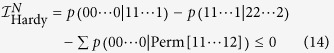


Here Perm[11 …12] is any permutation of indices between parties, and the summation is taken over all such permutations that are possible. We see that we have the following cases:

(i) One, or both, of 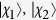 is entangled. If 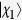 is entangled, one can use the following Bell inequality


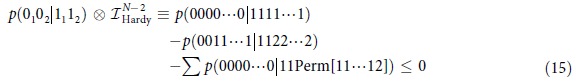


to detect violation of local realism. Here 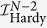 is the (*N* − 2)-qubit Hardy inequality for 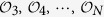. The validity of this inequality to test the violation of local realism of 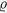 relies on the fact that when 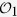 and 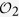 measure their qubits respectively along the *z*-direction, these measurements are equivalent to a test for the violation of local realism of 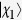 using 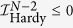. Also, if 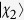 is entangled, the violation of local realism is detected in similar manner.

(ii) None of the states 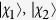 is entangled. Up to LU, one can work with 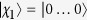 and 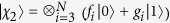, where *f*_*i*_ ≥ 0.

If the number of coefficients *f*_*i*_ that are equal to zero is *r* ∈ {0,1,…...,*N* − 3} (without loss of generality we have assumed that the first *r* coefficients are zero), then we use the following Bell inequality


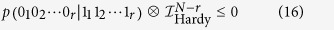


to test the violation of local realism. Let the settings be


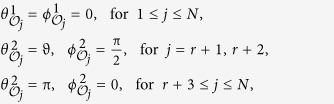


then quantum mechanics demands


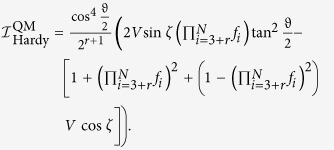


Similar to Eq. (7) in the *N* = 3 case in the main text and taking an appropriate value of *ϑ*, we always have 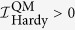.

If all *f*_*i*_ = 0 (i.e., *r* = *N* − 2), for an even *N*, the violation of local realism of the state 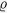 is dictated by


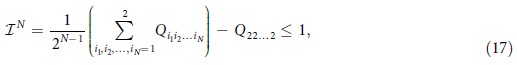


where correlation functions 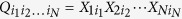, with 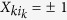, and *i*_*k*_ indicating settings for the *k*-th party. For *N* = 2, the inequality (17) reduces to the CHSH inequality.

The classical bound is obtained as follows. The summation term in (17) is the binomial expansion of 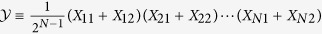, which could take only three distinct values {−2, 0, 2}. The value of *Q*_22 …2_ is closely related to 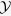. Indeed, when 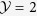, then *Q*_22 …2_ = 1 so that 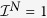; when 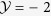, then *Q*_22…2_ = −1 so that 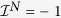; when 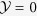, then 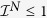 since *Q*_22….2_ is no larger than 1. Hence, 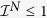 holds for any local theories.

In quantum mechanics, the correlation function is computed by 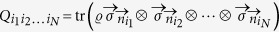, with 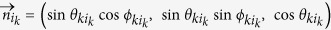. We explicitly have


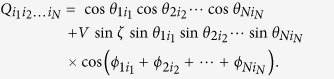


Taking the settings as 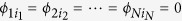, *θ*_*N*1_ = 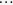 = *θ*_21_ = π − *θ*_11_, *θ*_*N*2_ = 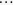 = *θ*_22_ = π − *θ*_12_, and *θ*_12_ = π, we get the quantum bound as


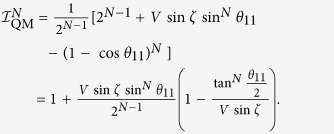


Clearly, we have 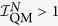 when 
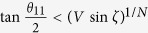
.

For odd *N*, violation of local realism of the state 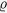 is identified by


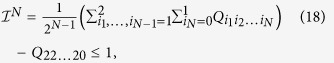


where *i*_*N*_ = 0 indicates that no measurement is performed on the *N*-th qubit. Quantum mechanically, we explicitly have


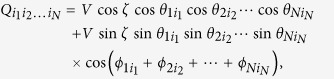


and, 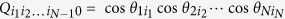. By taking settings as 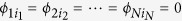, 
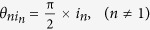
, and *θ*_12_ = 0, all correlation functions vanish except the following ones: *Q*_111 …11_ = *V*sin *ζ* sin*θ*_11_, *Q*_122…20_ = −cos*θ*_11_, and *Q*_222…20_ = −1. Hence, the quantum prediction of 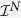 in (18) is


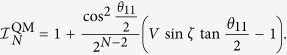


Clearly, we have 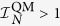 when 
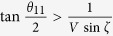
, proving Theorem 2.

## Additional Information

**How to cite this article**: Chen, J.-L. *et al.* Beyond Gisin's Theorem and its Applications: Violation of Local Realism by Two-Party Einstein-Podolsky-Rosen Steering. *Sci. Rep.* **5**, 11624; doi: 10.1038/srep11624 (2015).
